# “Hills of Activity” as an effective method for mitigating ground surface vibration in sustainable residential development

**DOI:** 10.1371/journal.pone.0332914

**Published:** 2025-09-29

**Authors:** Aneta Herbut

**Affiliations:** Wroclaw University of Science and Technology, Faculty of Civil Engineering, Institute of Geotechnics and Hydrotechnics, Wroclaw, Poland; Universite Cote d'Azur, FRANCE

## Abstract

This paper investigates the vibration mitigation efficiency of a hill and a system of hills. The effects of obstacle height, slope inclination angle, the number of embankments used, and their shape on vibration reduction levels are analyzed. It has been observed that larger, flatter hills reduce the dominant vertical velocity component more effectively than smaller, steeper ones. Multiple rows of obstacles can be employed to improve vibration reduction. The effectiveness of the solution was examined for both low-frequency (20 Hz) and high-frequency (60 Hz) excitations. The vertical component of the velocity vector may be reduced to as low as 30% of its initial value due to the application of the hill(s) or row(s) of hills. The main advantage of the proposed solution, in the form of a convex soil obstacle, is that it reduces both vibrations and noise (factors that often co-occur). Moreover, the system of hills can also function as playgrounds, providing a recreational space for children. Classical concave barriers (such as commonly used open trenches) do not offer such benefits. The proposed solution aligns with an important research direction in sustainable residential development.

## 1. Introduction

### 1.1 Research motivation

Although the concepts of “health” and “well-being” have long been central to medicine and psychology, their importance has extended into diverse fields, including architecture and engineering. Researchers globally are now examining how everyday environments affect individuals’ overall health, satisfaction, and quality of life [1]. Negative influences on our functioning include factors such as vibrations and noise, which are commonly identified as environmental pollutants [2]. Conversely, engaging in outdoor activities, particularly in social settings, has a demonstrably beneficial impact on both physical and mental health. Furthermore, urban and landscape greenery enhancement has been linked to improved health, increased well-being, and greater resilience [3]. As a result, areas such as “architecture of health & well-being,” “sustainable design,” “biophilic design” and “biophilic architecture” have emerged as crucial research directions in both architecture and civil engineering. This design methodology prioritizes human well-being by acknowledging the innate need to forge bonds with nature and its natural components. Detailed studies have confirmed that these connections provide notable benefits to both the physical and mental health of building occupants, including adjusted heart rhythms, lower stress levels, enhanced cognitive performance and improved mood [3–6]. These issues are particularly important for children and adolescents [4,7–10].

This paper examines the effect of landscape formation on the reduction of wave energy propagating through the ground. Man-made vibrations caused by construction activities, machinery, and highway and railway traffic may disturb adjacent structures and sensitive equipment. They may also bother residents, causing heightened stress, sleep disorders or irritability. Depending on the source of the vibrations, those induced by human activity exhibit different characteristics of the excitation force.

In the present study, the focus was placed on man-made vibrations caused by geotechnical works or vehicular traffic (with a dominant forcing frequency of approximately 20 Hz [11–12]). In addition, the effectiveness of the proposed solution was examined for a higher-frequency excitation, typical of areas located near subway or railway lines (with a dominant forcing frequency of approximately 60 Hz [13–14]).

The paper verifies the effectiveness of a system of elevations of different shapes in mitigating vibrations. Elevation as an acoustic baffle is a solution commonly used in practice [15]. Its effect on ground surface vibrations, however, has been much less studied. This is because convex rather than concave baffles in the soil medium are more commonly used – in the form of so-called wave barriers and trenches [16]. Ground obstacles built from elevations are not as widely applied.

**Fig 1:**
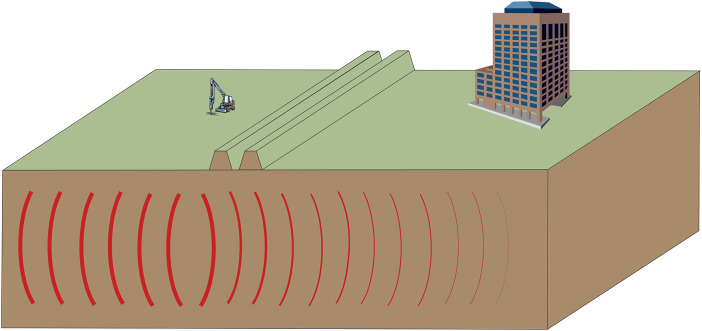
“Hills of Activity” designed to mitigate noise and vibration impacts on structures.

The inspiration for this paper comes from children’s playgrounds that have been built recently in Poland in the form of hill systems (“hills of activity”), such as those in “Park Ujazdowski” in Warsaw, “Park Wolności” in Pabianice, and children’s playgrounds in Sopot and Michałowice (Fig 1). If the arrangement of hills could serve not only to reduce noise (a known and applied solution) and vibrations (a factor usually associated with noise) but also to create a green screen and provide children with an area for play and recreation, the benefits would be even greater. A properly designed soil partition can be a place for active leisure and socialization for children and young people (e.g., slides, climbing walls, sledding hills, and skate parks). With the right choice of plants, it can have a beneficial effect on the senses of hearing and sight by separating the source of vibrations (e.g., streets, construction sites, factories) from residential and recreational areas (Fig 1).

### 1.2. Methods of reducing ground vibrations

There are two main approaches to protecting buildings and people from seismic and paraseismic vibrations [17]. The first involves adjusting construction parameters, such as stiffness and damping, by using various dampers and vibration absorbers [18–19]. The second approach reduces the energy of seismic waves before they reach the structure. This method can safeguard a wider area, protecting multiple buildings rather than just one. It employs different types of horizontal and vertical wave barriers placed between the source of vibration and the structure [16, 20–41]. When a seismic wave hits a ground barrier, part of its energy is reflected and refracted, creating new body waves. Both vertical [16,20–30] and horizontal [31–35] ground obstacles are common techniques for mitigating vibrations. Recent breakthroughs in photonic and phononic crystals have sparked a growing interest in reducing the energy carried by waves propagating in soil. Consequently, using metamaterials in the form of wave barriers and periodic structures (or foundations) shows great promise for shielding structures and people from the destructive effects of earthquakes and man-made vibrations [42–49].

A straightforward and widely used method for minimizing ground vibrations involves installing a vertical barrier in the soil between the source of vibrations and the area to be protected. Woods was the first to present extensive experimental results on this technique [16]. His studies on the efficiency of open (empty) trenches – tested under a vertical harmonic load applied to the ground surface – revealed that the vibration reduction improves when the barrier is positioned closer to the vibration source and its depth is more than about 60% of the Rayleigh wavelength (λ_R_) [16]. In other words, vertical ground barriers can effectively mitigate vibrations, but they work best when the Rayleigh wavelength is relatively short, which typically occurs in softer soils near the surface and/or under high-frequency excitations. When comparing barriers with the same geometric and mechanical properties, empty trenches (often called “open trenches”) tend to offer better vibration reduction than filled ones [20–23]. However, open trenches can encounter issues such as slope instability or groundwater ingress, problems that are generally alleviated by using filled trenches. By introducing a material with properties significantly different from the surrounding soil, the surface waves are reflected and refracted, effectively scattering the wave energy.

It is not always feasible to construct a vertical barrier in the soil, and sometimes this approach is not adequately effective. Alternatively, horizontal barriers, typically situated near, beneath the vibration source, are commonly employed to safeguard buildings, individuals, and sensitive equipment in the vicinity of railway tracks or machinery. Chouw et al. demonstrated that a rigid layer placed at a shallow depth can attenuate harmonically excited vibrations for frequencies below a certain threshold [31–32]. Horizontal rigid ground obstacles can be more effective than vertical trenches with identical dimensions, particularly at lower excitation frequencies [32,34].

In a more general approach, rather than assuming a vertical or horizontal orientation for the partition, the problem of selecting its geometric (such as location and inclination) and material parameters was solved using optimization algorithms [36–37]. The authors showed that factors such as the inclination of the partition, its volume, or the depth at which it is buried can significantly affect the solution’s effectiveness [36]. The study [36] was conducted through numerical analyses using the finite element method for both dry and saturated media. Stiff barriers in the form of single and double walls were investigated by Bordón et al. over a wide range of excitation frequencies [37]. The influence of the barrier position, inclination, length, and thickness on the efficacy of vibration mitigation was investigated. The authors stated that these barrier topologies may improve the vibration reduction effect, especially for obstacles located at a depth greater than the Rayleigh wavelength (λ_R_) [37].

Over the past few years, researchers have been exploring the use of waste and environmentally friendly materials to fill both vertical [38–40] and horizontal barriers [41]. Materials like tire-derived aggregates [38,41], wood shavings, Styrofoam, or expanded glass granules [40] maintain the vibration mitigation benefits while also offering a positive environmental impact. Moreover, recent studies on wave barriers have increasingly focused on the use of periodic structures, specifically in the form of seismic metamaterials [42–49]. The design of these barriers is pursued along two primary paths: one aims to achieve optimal wave scattering (commonly known as “Bragg scattering”), while the other focuses on dissipating wave energy through resonant mechanisms (“local resonances”). Metamaterials developed for seismic protection via optimal scattering consist of numerous hollow or solid inclusions embedded within the soil matrix, strategically located between the vibration source and the structure [42 44]. By carefully selecting the elastic moduli and densities of both the matrix and the inclusions, it is possible to create an effective barrier against surface waves. When these waves encounter the inclusions, they are reflected and refracted, resulting in a reduction in their energy. Alternatively, embedding specialized elements within the soil matrix can trigger resonant vibrations in the inclusions – a phenomenon known as “local resonances” – thereby dissipating part of the wave energy [45–48]. Owing to the periodic arrangement of inclusions within the soil, this type of barrier is also referred to as a metabarrier. Moreover, the two approaches can be combined to further enhance the barrier’s effectiveness in mitigating both high and low frequencies [49].

## 2. Mathematical and numerical model

The equations of motion for the axisymmetric state of stress in cylindrical coordinates can be written as:


$$\frac{\partial \sigma_{r}}{\partial r}+\frac{\partial \tau_{zr}}{\partial z}+\frac{\sigma_{r}-\sigma_{\theta }}{r}=\rho \frac{{\partial^{\text{2}}u}_{r}}{\partial t^{\text{2}}},\;\frac{\partial \tau_{rz}}{\partial r}+\frac{\partial \sigma_{z}}{\partial z}+\frac{\tau_{rz}}{r}=\rho \frac{{\partial^{\text{2}}u}_{z}}{\partial t^{\text{2}}}.$$ (1)

Substituting strain-displacement relationships: $\varepsilon_{r}=\frac{\partial u_{r}}{\partial r},\;\varepsilon_{z}=\frac{\partial u_{z}}{\partial z},\;\tau_{rz}=\frac{\text{1}}{\text{2}}\left(\frac{\partial u_{z}}{\partial r}+\frac{\partial u_{r}}{\partial z}\right)$ and stress-strain relationships: $\sigma_{r}=(\lambda +\text{2}\mu )\varepsilon_{r}+\lambda \varepsilon_{z},\;\sigma_{z}=\lambda \varepsilon_{r}+(\lambda +\text{2}\mu )\varepsilon_{z},\;{\sigma_{\theta }=\lambda \left(\varepsilon_{r}+\varepsilon_{z}\right),\;\tau }_{rz}=\text{2}\mu \varepsilon_{rz}$ into equation (1) yields the final form of the Navier-Lamé equations for the axisymmetric state of stress in cylindrical coordinates:


$$(\lambda +\mu )\frac{\partial }{\partial r}\left(\frac{\text{1}}{r}\frac{\partial \left(ru_{r}\right)}{\partial r}+\frac{\partial u_{z}}{\partial r}\right)+\mu \left(\nabla^{\text{2}}u_{r}-\frac{u_{r}}{r}\right)=\rho \frac{{\partial^{\text{2}}u}_{r}}{\partial t^{\text{2}}},(\lambda +\mu )\frac{\partial }{\partial z}\left(\frac{\text{1}}{r}\frac{\partial \left(ru_{r}\right)}{\partial r}+\frac{\partial u_{z}}{\partial z}\right)+\mu \nabla^{\text{2}}u_{z}=\rho \frac{{\partial^{\text{2}}u}_{z}}{\partial t^{\text{2}}},$$ (2)

where *u_r_*, *u_z_* are displacements in radial and vertical directions respectively; λ, μ are the Lamé constants ($\lambda =\frac{E\vartheta }{\left[(\text{1}+\vartheta )(\text{1}-\text{2}\vartheta )\right]}$; $\mu =\frac{E}{\left[\text{2}(\text{1}+\vartheta )\right]}$, where E is the Young modulus and ϑ is the Poisson ratio); ρ is the density of the elastic medium; $\nabla^{\text{2}}=\frac{\partial^{\text{2}}}{\partial r^{\text{2}}}+\frac{\text{1}}{r}\frac{\partial }{\partial r}+\frac{\partial^{\text{2}}}{\partial z^{\text{2}}}$ is the Laplacian operator [50–51].

**Fig 2:**
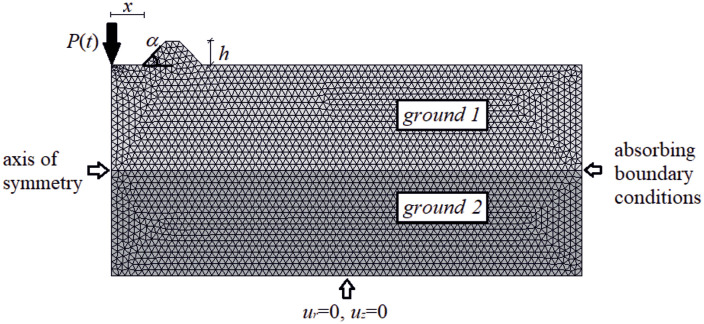
Analyzed numerical model and assumed symbols.

To avoid wave reflection from the boundaries of the analyzed region, absorbing boundary conditions, as proposed by Lysmer and Kuhlemeyer [52], are employed. The normal (σ_r_) and shear (τ_rz_) stress components for virtual dampers attached to the right (along coordinate r = 70m in Fig 2) boundary are given by $\sigma_{r}=a\rho V_{P}v_{r}$, $\tau_{rz}=b\rho V_{S}v_{z}$, where v_r_, v_z_ are components of the velocity vector in radial and vertical directions respectively; V_S_ denotes the S-wave velocity and V_P_ denotes the P-wave velocity. Research findings indicate that setting a = 1 and b = 0.25 yields reasonable absorption at the boundary [43]. Both displacement components are assumed to be zero at the bottom edge of the investigated region, i.e., u_r_ = u_z_ = 0. Harmonic force P(t) is applied to the concrete plate located in the left part of the analyzed area (Fig 2). The amplitude of the excitation force is A = 30kN, the frequency is f = 20 Hz. The layered soil medium is assumed in the analyses with the following parameters: E_1_ = 40MPa, ν_1_ = 0.3, ρ_1_ = 1700 kg/m^3^ (ground layer 1, Fig 2); E_2_ = 80MPa, ν_2_ = 0.3, ρ_2_ = 1800 kg/m^3^ (ground layer 2, Fig 2). A fully bonded soil plate interface is assumed. The maximum size of the grid elements is limited, according to Kramer, to one-fifth of the minimum wavelength considered in the analysis [53].

## 3. Results

To compare the results obtained before and after applying the wave barrier, a commonly used nondimensional factor, the amplitude mitigation factor (AMF), is introduced [9]. The amplitude mitigation factor, denoted as AMFv_i_ (for i = r, z) for velocities in both the radial and vertical directions is expressed as


$${AMFv}_{i}=\frac{Max\left(|\;v_{i}|\;\right)}{Max\left(|\;v_{i,o}|\;\right)}.$$ (3)

**Fig 3:**
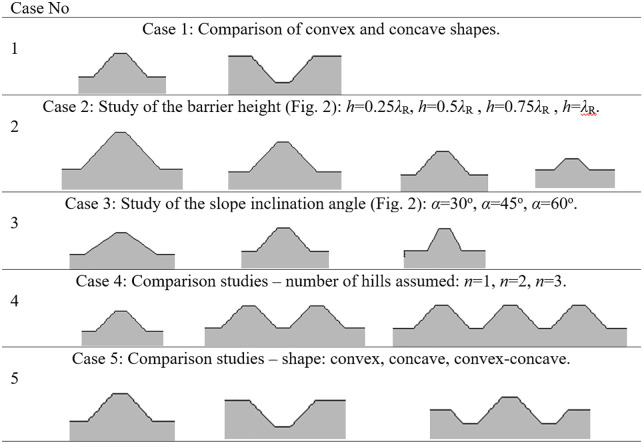
Shapes of the wave barriers analyzed in the paper.

Calculations are performed for two cases: with the wave obstacle (where v_i_ – velocity component in i-direction) and without the wave obstacle (where v_i,o_ – velocity component in i-direction). The maximum values of the velocities are compared for both cases. Velocity components are analyzed instead of displacement components, because most standards for vibration monitoring of structures [54 57] rely on velocity measurements. For both situations (with and without the wave obstacle), the maximum absolute values of the velocity components are established. Calculations are performed in the time domain (from 0 to 11T, where T = 1/f is the period of the excitation force) for 20 uniformly distributed points along the ground surface. For each point, the AMF is evaluated. The assumed analysis duration of 11T is sufficient for the surface and body waves to cover the distance between the excitation force P(t) (at r = 0, z = 0) and the boundary of the analyzed region (at r = 70m, z = 0). The appropriate partial differential equations are solved using FlexPDE Professional V6 software (www.pdesolutions.com). Then, the results are analyzed using Mathematica 11 software (www.wolfram.com). Various shapes of the convex ground obstacle are examined in this study to verify the solution’s effectiveness and to select the most efficient design (Fig 3).

**Fig 4:**
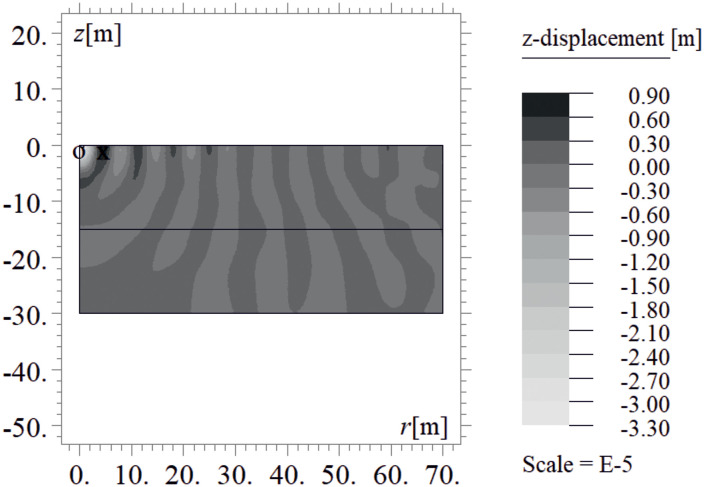
Wave propagation in the ground – vertical component of the displacement vector at t = 0.48 s, without a wave obstacle.

**Fig 5:**
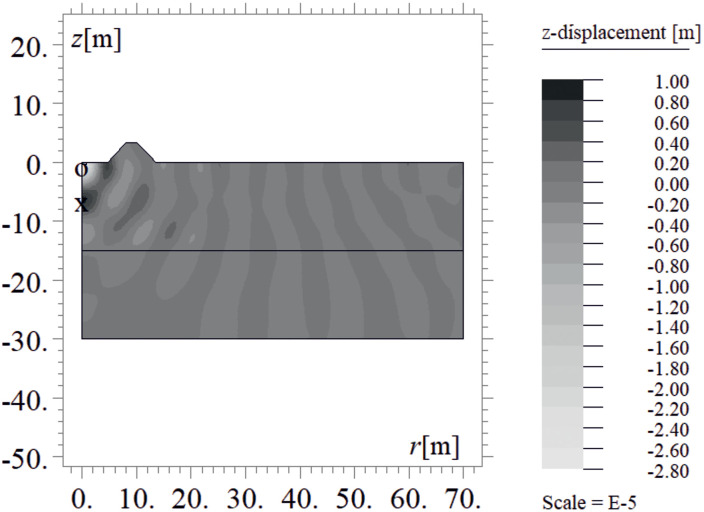
Wave propagation in the ground – vertical component of the displacement vector at t = 0.48 s, with a wave obstacle.

**Fig 6:**
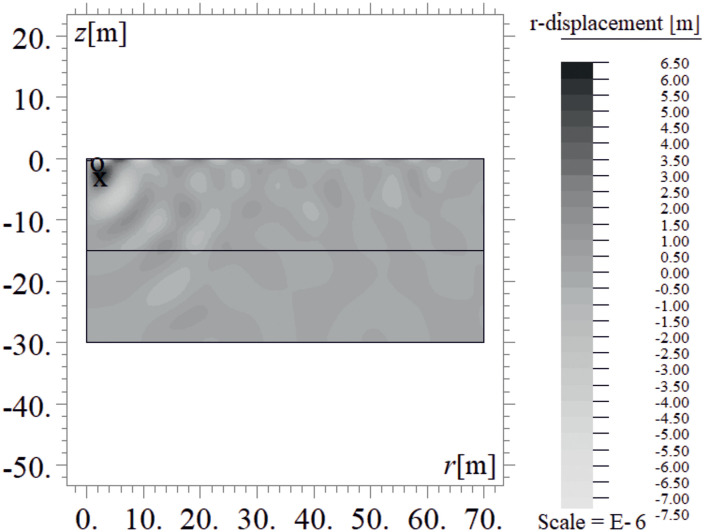
Wave propagation in the ground – radial component of the displacement vector at t = 0.48 s, without a wave obstacle.

**Fig 7:**
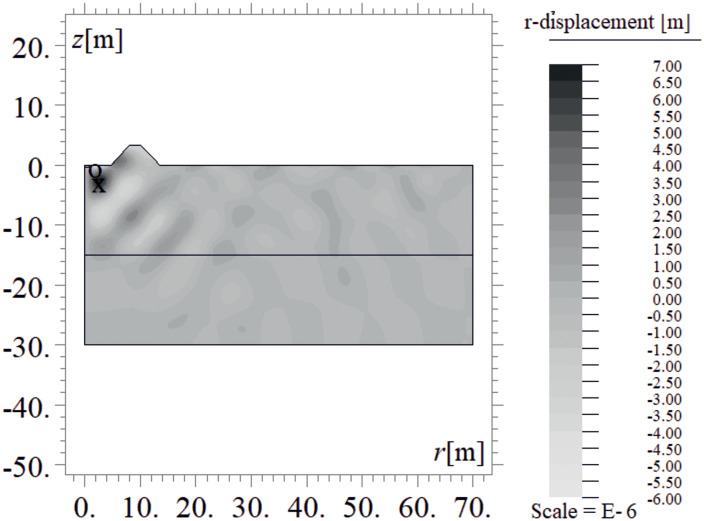
Wave propagation in the ground – radial component of the displacement vector at t = 0.48 s, with a wave obstacle.

**Fig 8:**
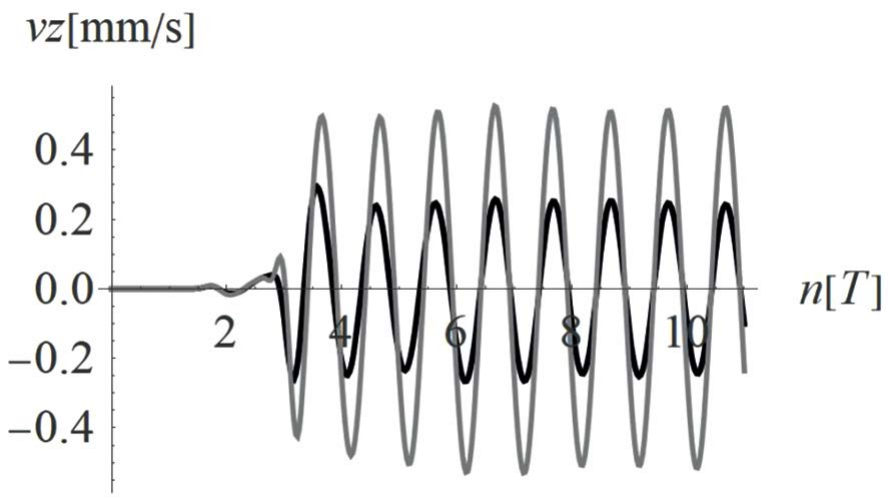
Vertical components of the velocity vector with (black) and without (gray) a single convex wave obstacle (see Fig. 2) for a selected point on the ground surface located at a distance of 24.5 m from the vibration source.

**Fig 9:**
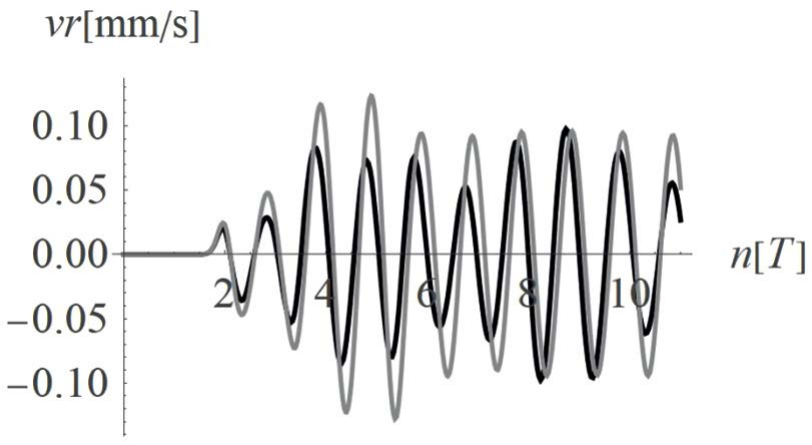
Radial components of the velocity vector with (black) and without (gray) a single convex wave obstacle (see Fig. 2) for a selected point on the ground surface located at a distance of 24.5 m from the vibration source.

Due to the harmonic load applied to the ground surface (Figs 4–7), both surface and body waves are generated. Rayleigh waves spread out with a cylindrical wavefront, while body waves propagate within the soil medium. It can be observed that the velocity component in the vertical direction is approximately four times higher than the radial component (Figs 8–9), which is important for further analyses. From the standpoint of structural safety and occupant comfort, the peak particle velocity (PPV) is a critical parameter. Threshold values of PPV are defined in international codes for vibration monitoring to determine building safety levels [54–57]. PPV is the maximum value of the two components of the velocity vector – vertical and radial (with the radial component measured along the line between the structure and the vibration source). In the analyzed cases, the vertical component was always dominant; therefore, it should receive primary attention. As shown in Figs 4–9, the convex wave obstacle (as depicted in Fig 2 – with h = 0.5λ_R_, α = 45^o^, x = 4m) reduces both components of the velocity vector; however, the reduction in the vertical component is greater (AMF_vz_ ~ 0.5). The vibration reduction effect observed after contact with a convex or concave barrier is caused by wave energy scattering — specifically, by the phenomenon of wave reflection from the barrier edges. By altering the direction of propagation, part of the wave energy is directed into the ground as well as back toward the vibration source [Figs 4–7].

**Fig 10:**
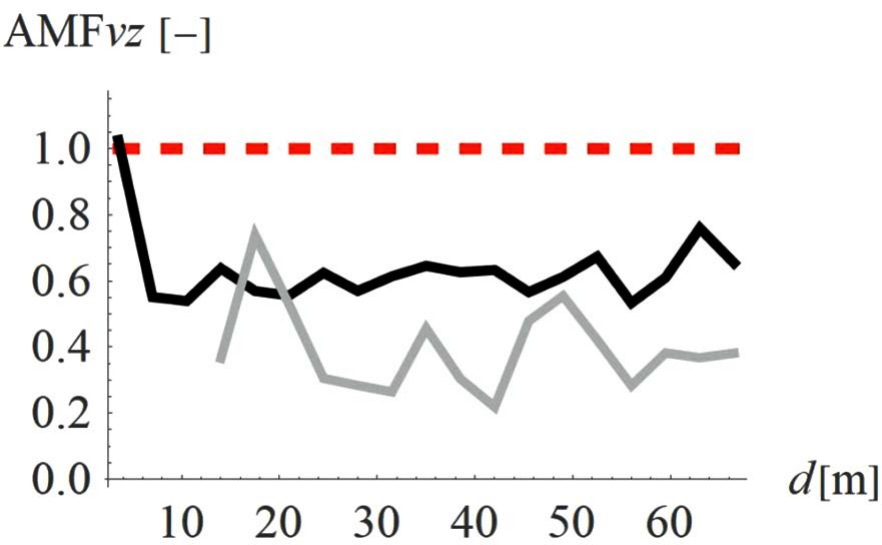
AMF for the vertical velocity vector components for two different shapes – convex (gray) and concave (black).

**Fig 11:**
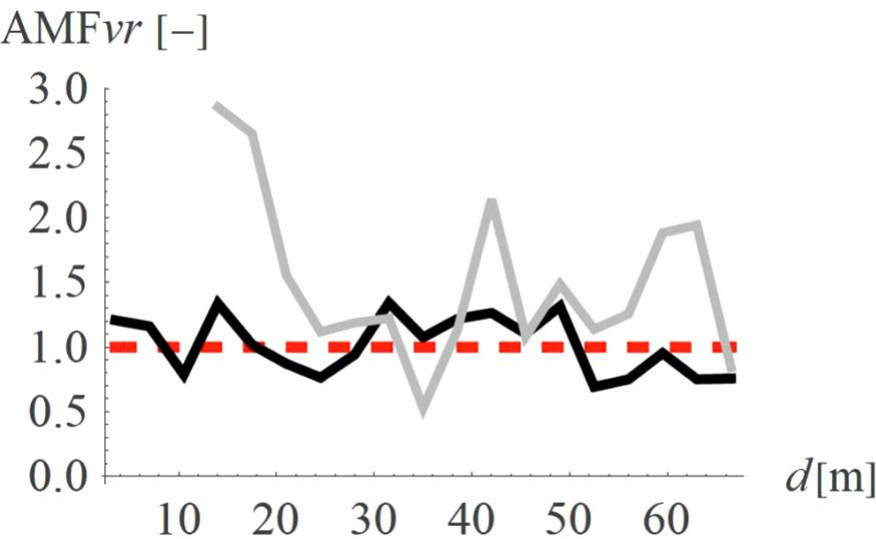
AMF for the radial velocity vector components for two different shapes – convex (gray) and concave (black).

Holes (concave shape, so called “open trenches”) are more common solutions than hills (convex shape). Figs 10–11 present a comparison between these two shapes is terms of AMF_vz_ (Fig 10) and AMF_vr_ (Fig 11). The red dashed line shows the boundary between the effect of vibration amplification (AMF_vi_ > 1) and vibration reduction (AMF_vi_ < 1). It is evident that the concave shape provides a better vibration reduction effect for the predominant vertical velocity component (Fig 10); however, for the horizontal component the effect is reversed (Fig 11). Despite this hills can still give a satisfactory reduction effect for the vertical velocity component (AMF_vz_ = 0.61). The advantage of the hills constructed on the ground surface compared to the holes is that they reduce not only vibrations but also noise. Moreover, for soil trenches, many problems may appear like slope stability and groundwater flows into the hole. This terrain also poses a danger to animals, which may have difficulty escaping the pit due to the relatively steep slopes. These issues do not occur in the case of hills, which can additionally serve a recreational function (activity hill, playground, sledding hill, skate park, etc.).

**Fig 12:**
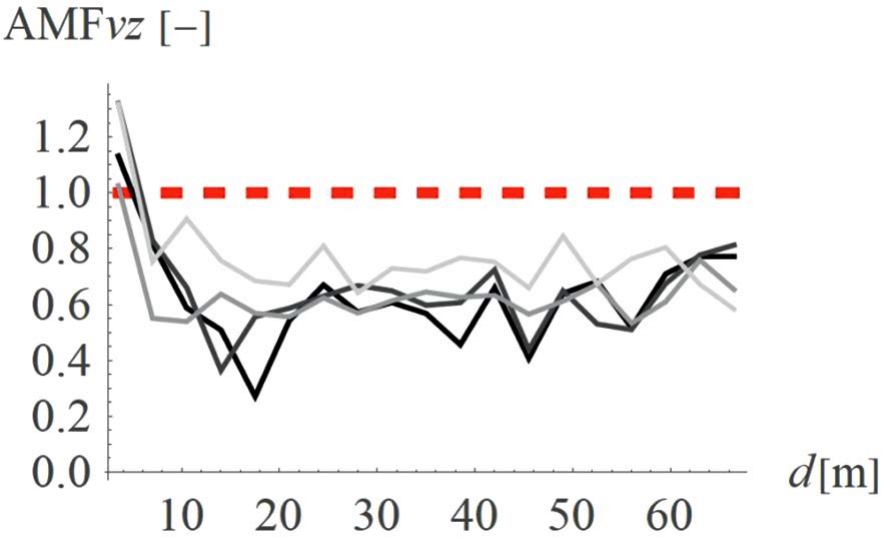
AMF for the vertical velocity vector components for different hill heights: h = λ_R_ = 6.7m (black), h = 0.75λ_R_ = 5.0m (dark gray), h = 0.5λ_R_ = 3.6m (gray), h = 0.25λ_R_ = 1.7m (light gray).

**Fig 13:**
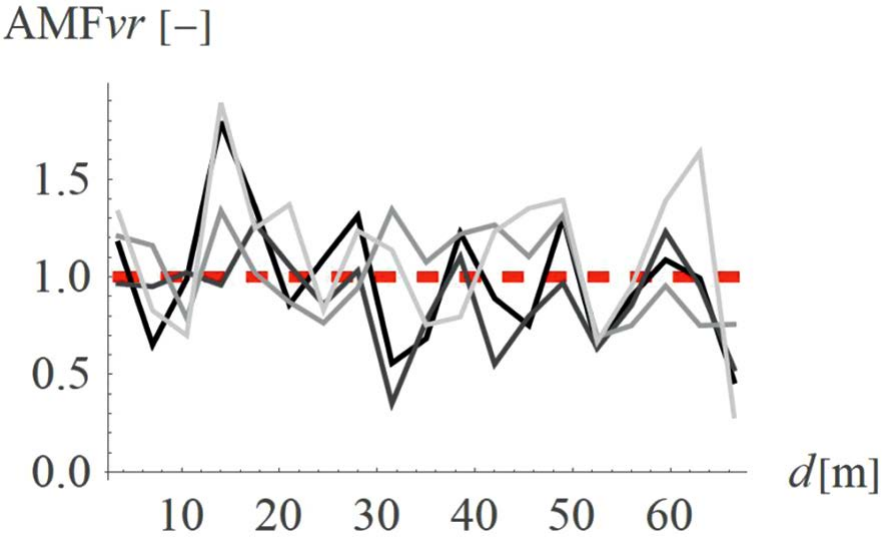
AMF for the radial velocity vector components for different hill heights: h = λ_R_ = 6.7m (black), h = 0.75λ_R_ = 5.0m (dark gray), h = 0.5λ_R_ = 3.6m (gray), h = 0.25λ_R_ = 1.7m (light gray).

For concave wave barrier shapes (“open trenches”), the obstacle height is generally one of the most significant factors influencing the vibration mitigation efficiency [16]. The question is whether the same effect holds true for convex obstacle shapes. To answer this question, four different obstacle heights were analyzed (Case 2 in Fig 3). The results in the form of AMF for two components of the velocity vector are presented in Figs 12–13. It can be seen that the obstacle height influences vibration mitigation efficiency similarly for both convex and concave shapes. In general, the higher the barrier, the more significant the vibration reduction effect on the critical vertical velocity component (Fig 12). The average values of AMF_vz_ for points located behind the wave obstacle are as follows: AMF_vz,0.25λR_ = 0.72; AMF_vz,0.5λR_ = 0.62; AMF_vz,0.75λR_ = 0.65; AMF_vz,λR_ = 0.58. For horizontal components, no similar regularity is seen (Fig 13). It should be noted, however, that when assessing the safety of the structure, the larger of the two components is decisive – in this case, the vertical component. A slight increase in vibration in the radial direction is not problematic, as this component is several times smaller than the vertical one (see Fig 8 and Fig 9).

**Fig 14:**
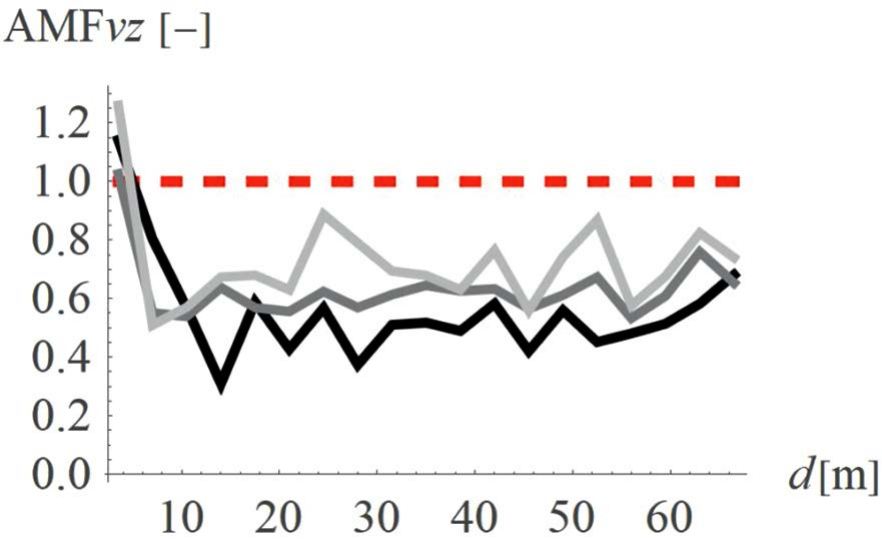
AMF for the vertical velocity vector components for different hill slope inclinations: α = 30° (black), α = 45° (gray), α = 60° (light gray).

**Fig 15:**
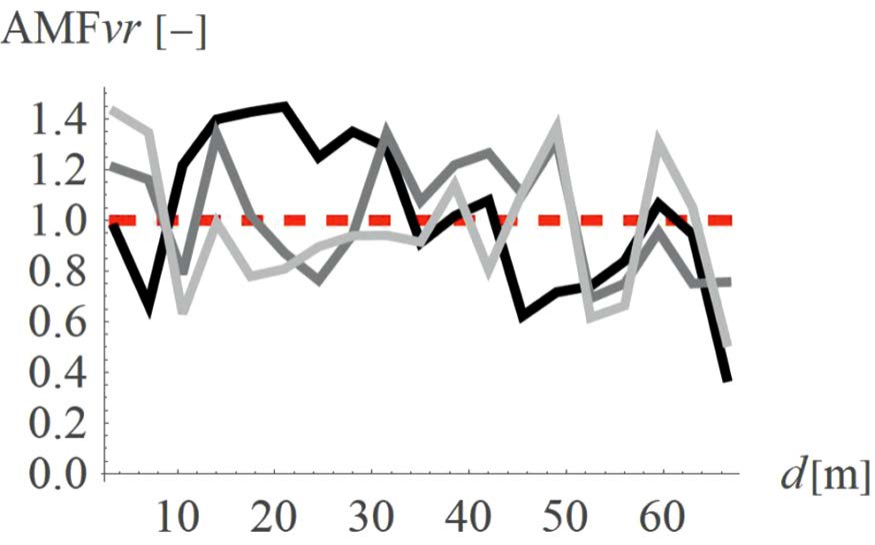
AMF for the radial velocity vector components for different hill slope inclinations: α = 30° (black), α = 45° (gray), α = 60° (light gray).

To verify how the slope inclination influences the vibration reduction level, four different hill shapes were analyzed (Case 3 in Fig 3). It can be seen that the smaller values of AMF_vz_ are achieved for a flat slope (α = 30^o^, black line in Fig 14, AMF_vz,α30_ = 0.51), compared to steep slope (α = 60^o^, fair gray line in Fig 14, AMF_vz,α60_ = 0.71). For the radial component of the velocity vector, such a conclusion cannot be drawn – both local amplifications and mitigations can be seen for each inclination angle (Fig 15). However, this component, similar to the previous case (Case 2) does not determine the safety and comfort of the structure’s use. Additional factors were verified during the research – a negligible effect of the width of the embankment and its location on the level of vibration reduction was observed.

**Fig 16:**
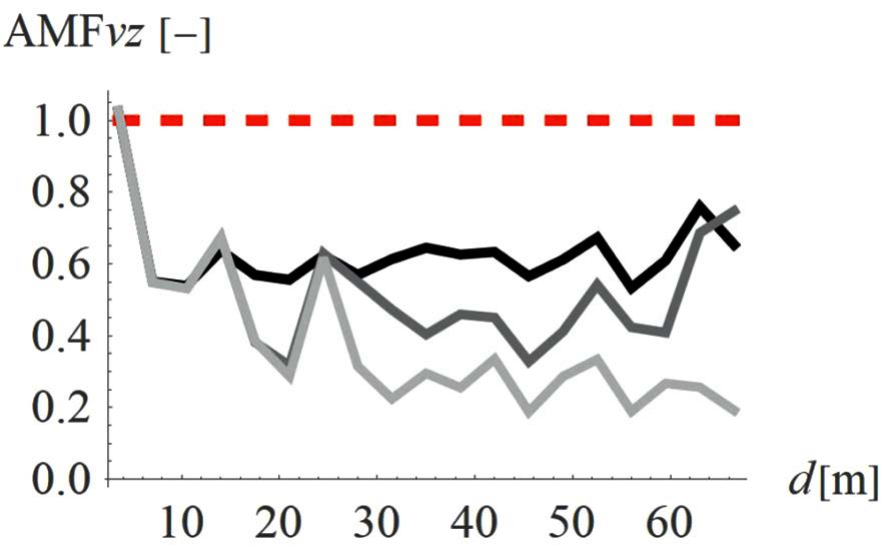
AMF for the vertical velocity vector components for different numbers of hills: n = 1 (black), n = 2 (gray), n = 3 (light gray).

**Fig 17:**
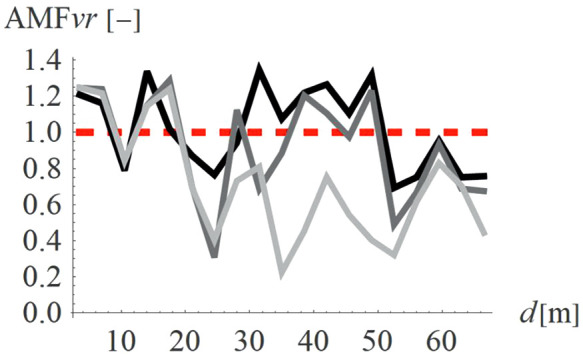
AMF for the radial velocity vector components for different numbers of hills: n = 1 (black), n = 2 (gray), n = 3 (light gray).

**Fig 18:**
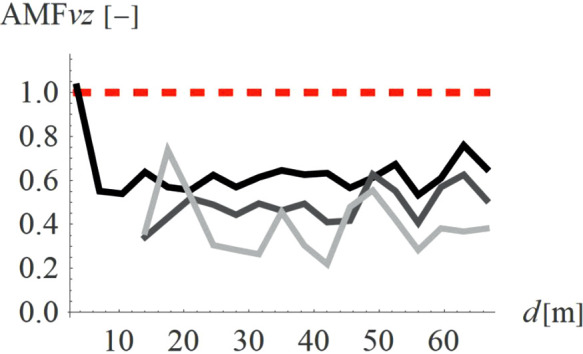
AMF for the vertical velocity vector components for different shapes of the ground obstacles: convex (black), concave (light gray), convex-concave (gray).

**Fig 19:**
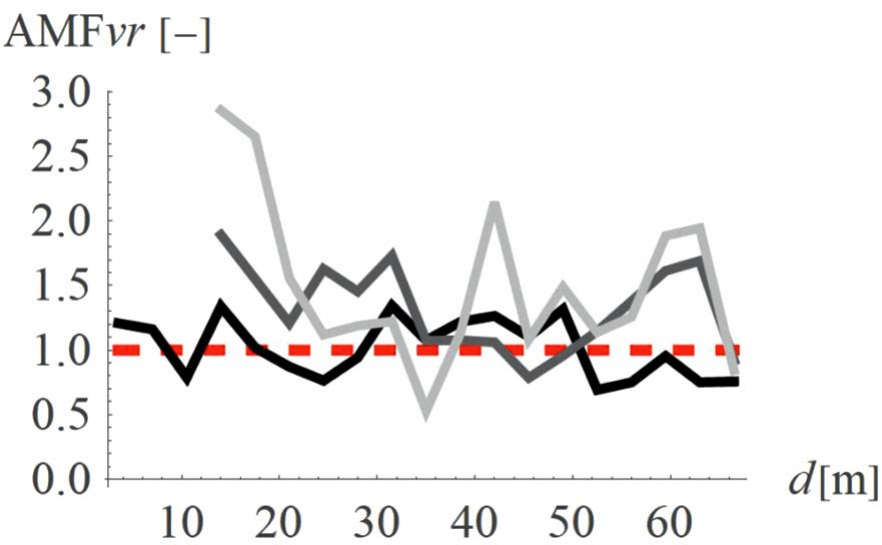
AMF for the radial velocity vector components for different shapes of the ground obstacles: convex (black), concave (light gray), convex-concave (gray).

To address the question of how to further increase the efficiency of the solution, two additional cases were studied – convex barriers located in several rows (Case 4 in Fig 3) and a concave-convex wave obstacle (Case 5 in Fig 3). The results for the selected examples are presented in Figs 16–17 (Case 4) and Figs 18–19 (Case 5). Both approaches may improve the vibration reduction effect. The most significant improvement in the efficiency of the solution can be observed when several rows of elevations are used (Figs 16–17). The effect of vibration reduction in this case is visible for both the vertical and horizontal components. For n = 1 the average value of AMF_vz_ for points located behind the obstacle is 0.62, whereas for n = 3 it is 0.29. The convex-concave shape of the obstacle (see Case 5 in Fig 3) can also improve the vibration reduction efficiency – AMF_vz_ = 0.61 for the convex shape of the obstacle, AMF_vz_ = 0.50 for the convex-concave shape and AMF_vz_ = 0.40 for the concave shape (Figs 18–19).

**Fig 20:**
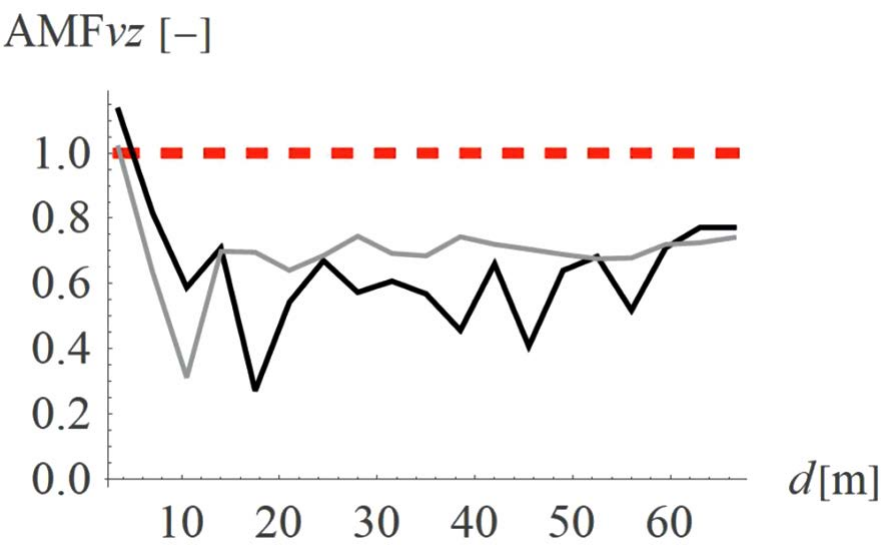
AMF for the vertical velocity vector components for a convex obstacle with h = λ_R_, at two different excitation frequencies – 20 Hz (black) and 60 Hz (gray).

**Fig 21:**
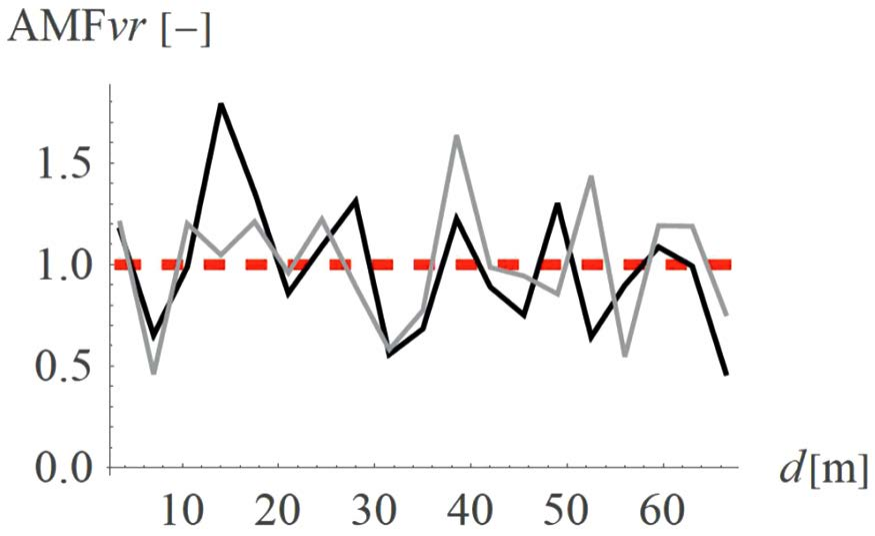
AMF for the radial velocity vector components for a convex obstacle with h = λ_R_, at two different excitation frequencies – 20 Hz (black) and 60 Hz (gray).

**Fig 22:**
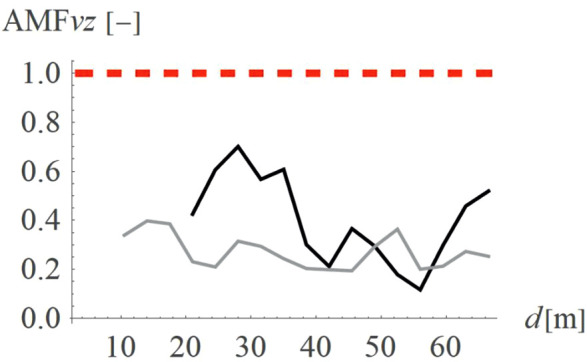
AMF for the vertical velocity vector components for a concave obstacle with h = λ_R_, at two different excitation frequencies – 20 Hz (black) and 60 Hz (gray).

**Fig 23:**
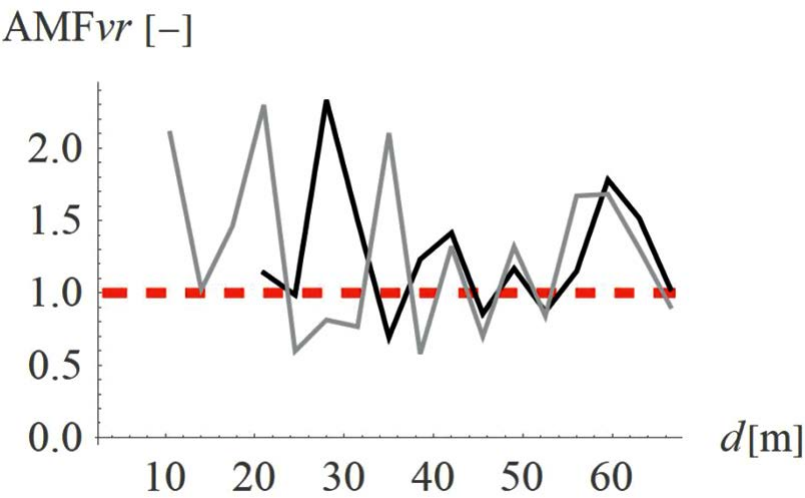
AMF for the radial velocity vector components for a concave obstacle with h = λ_R_, at two different excitation frequencies – 20 Hz (black) and 60 Hz (gray).

The results presented thus far pertain to excitation forces with a dominant excitation frequency of 20 Hz, representative of geotechnical works and dynamic effects induced by road traffic [11–12]. In contrast, loads generated by rail transport are characterized by substantially higher excitation frequencies, typically in the range of 50–60 Hz [13–14]. In order to draw broader conclusions, the effectiveness of the proposed solution was investigated for an excitation frequency of 60 Hz (Figs 20–23). Figs 20–21 present AMF for the case of a single convex obstacle, whereas Figs 22–23 concern a single concave barrier (Fig 3, Case 1). In the analyzed cases, the barrier height was set equal to the wavelength of the surface wave. The results demonstrate a high efficiency of the proposed solution at higher excitation frequencies, with significant reductions observed mainly for the vertical component of the velocity vector. It should be noted that a barrier of the same height will be always more effective at higher excitation frequencies (i.e., shorter wavelengths). An analogous effect is observed for “open-trench” type barriers [16].

## 4. Conclusions

The paper presents a single hill and a system of hills as an effective solution for protecting structures, their inhabitants, and sensitive equipment against excessive man-made vibrations. Wave obstacles are commonly used to scatter wave energy on the path between the vibration source and the protected area. Proposals discussed in the literature deal mainly with concave obstacles, especially empty wave barriers (so-called “open trenches”), as an obvious baffle in the path of surface wave propagation. In the paper, the research focuses on convex obstacles (hills), which are less recognized in the literature. Elevations can have several advantages over concave obstacles, such as avoiding problems with groundwater inflow into the excavation and not posing a risk to animals. The paper pays special attention to aspects of sustainable residential development. This is because the layout of hills can serve not only as a tool for protection against vibrations and noise but also for screening industrial areas or transportation routes from residential areas. Vegetation-based shading undoubtedly improves residents’ comfort. In the paper, the possibility of using such a landscaped area as a place of recreation for children and young people (hills of activity) is emphasized.

Different shapes of the hills are analyzed in the paper to identify factors that can enhance the vibration reduction effect. The level of dynamic safety of the structure and the comfort of its use are determined by PPV (the peak particle velocity). PPV usually refers to the largest of the components of the velocity vector [44–57]. In the analyzed examples, the vertical component is several times higher than the radial one – hence, its level of reduction is decisive in the analyzed task. Slight amplification of the horizontal component is not relevant to the safety of structures and the comfort of their use.

During the analyses, it was observed that high, more flat elevations are more efficient than low, steep ones. Additionally, to improve the vibration mitigation effect, a concave-convex shape of obstacles is recommended. The most significant vibration reduction effect can be achieved by the use of several hills rather than a single one.

## Supporting information

S1 Data (NB)
